# Innovation in Chinese internet companies: A meta-frontier analysis

**DOI:** 10.1371/journal.pone.0233278

**Published:** 2020-05-21

**Authors:** Sadaf Hafeez, Noreen Izza Arshad, Lukman Bin A. B. Rahim, Muhammad Farooq Shabbir, Jawad Iqbal

**Affiliations:** 1 Department of Computer and Information Science, Universiti Teknologi PETRONAS, Seri Iskandar, Malaysia; 2 Department of Management Science, The Islamia University of Bahawalpur, Bahawalpur, Pakistan; Institute for Advanced Sustainability Studies, GERMANY

## Abstract

The innovation of a particular company benefits the whole industry when innovation technology transfers to others. Similarly, the development and innovation in internet companies influence the development and innovation of the industry. This investigation has applied a unique approach of meta-frontier analysis to estimate and analyze the innovation in internet companies in China. A unique dataset of Chinese internet companies from 2000 to 2017 has been utilized to estimate and compare the innovation over the period of study. The change in technology gap ratio (TGR) and a shift in production function have translated into innovation which was overlooked by previous studies. It is found that the production function of internet companies is moving upward in the presence of external factors such as smartphones invention, mobile internet, mobile payments, and artificial intelligence, etc. Consequently, a sudden increase in TGR is captured due to the innovation of some companies. Hence, the average TE of the industry falls caused by the increased distance of other companies form industry production function. However, the innovation advantage defused when other companies start imitating and the average TE elevates. A steady increase in the TGR index revealed that the continuous innovation-based growth of some companies lifting the production frontier upward. This provides the opportunity for other companies to imitate and provides continuous growth in the industry. This study provides a novel methodological approach to measure innovation and also provide practical implication by empirical estimation of innovation in Chinese internet companies.

## Introduction

The economic landscape has changed rapidly with the emergence of the ICT industry. This shift in the ICT paradigm has converged internet companies as an alternative investment for venture capitalists. The tremendous growth in internet companies has been witnessed with the emergence of the internet. Their increased digital participation not only adds new momentum to the economy but also changes the nature of growth.

Due to the highly competitive nature of this industry, companies focus on innovation to gain competitive advantage. Innovation is not only significant to achieve organizational success but important for the development of the industry. Innovation worked as a key element to grasp entrepreneurial opportunities. The process of innovation is not only changing the business and industry dynamics but it helps the business to perform according to social and environmental needs [1]. Innovation comes in different ways that generally lead to improved products and services, improved production technology and improved forms of firms and organizations. However, in this new ICT ecosystem era, the firms are innovating to improve products and services and adopting better technologies to improve production processes. Earlier studies have drawn a significant role of innovation in sustainable business development using efficiency dynamics [2,3]. Sustainable innovation-based business models considered as a key driver for business development in the ever-changing ICT landscape [4]. It is further argued that innovation generally does not last long and the first-mover advantage defused by imitation [5]. Nevertheless, the process of innovation and imitation is significant to improve overall productivity and provide growth opportunities [6]. Hence, it is worth investigating the role of innovation and imitation on the efficiency of the overall industry of internet companies. This will help to understand whether the growth and development of Chinese internet companies are due to internal innovation or external factors that stimulate growth.

As a member of the information and communication technology ecosystem, Internet companies do not develop and innovate on their own [7]. The convergence of PC to tablets and smartphones with advanced computing techniques provided opportunities for internet companies to innovate as a member of the ICT ecosystem. The interdependence of the ICT sector is increasing with technology innovation. The mobile application can track and monitor user activities to offer location-based services. The connected mobile device is becoming more important than ever. Hence, the changing landscape of the ICT industry made it difficult for an ICT firm to build all the necessary capabilities to compete in the market. Similarly, Fransman [8,9] argued that the ICT ecosystem has a co-evolutionary mechanism due to the increasing interdependence of ICT companies. The widespread use of the Internet has transformed the ICT ecosystem into a four-tier, evolving innovation system of ICT (i) hardware providers, (ii) network providers, platforms, (iii) content and application providers and (iv) end-users. Innovation is generated by the symbiotic interaction between the four layers in the ICT environment [8–12]. It is therefore essential to understand the role of the ICT ecosystem at the national level to promote open innovation and cooperation among enterprises [13,14].

Therefore, to understand the changes in the ICT industry in general and within internet companies in particular, this study is stimulated to empirically investigate the innovation in internet companies and the role of the ICT ecosystem in the innovation of internet companies. Moreover, this investigation correlates the external events in the ICT industry with innovation events in internet companies. Previous studies have drawn their attention to measuring the importance of innovation in firms and the role of ICT in the innovation of companies [15] while ignored the innovation in ICT companies particularly the internet companies. A dearth of literature has been found on internet companies in terms of innovation. Moreover, prior studies focused to measure the performance of internet companies using efficiency estimation approaches like DEA and SFA [16–18]. However, no such study has found to investigate the innovation in internet companies particularly using the ICT ecosystem perspective. Therefore, this study proposes to analyze the innovation of internet companies within the National ICT ecosystem as the National ecosystem affects the efficiency of individual firms. Furthermore, this study contributes to the body of knowledge by using a distinctive methodological approach to measure innovation. Earlier studies utilize the production function approach to measure the efficiency of a particular industry/group of companies where an increase in production function corresponds to the overall increase in industry productivity and a decrease in production function is understood as an overall decline in the productivity of industry and/or group [16,18,19]. Whereas, this investigation interprets the decline in production function is due to innovation which is measured by the technology gap ratio.

Over the past decade, internet companies have become a big industry in China. A massive increase in capital investment for sustainable innovation has been witnessed in recent times [20]. A huge investment in innovation-based RND projects by Chinese companies in the ICT sector has been witnessed in recent decades resulted in world-class internet companies [19]. These facts create the need to study how Chinese internet companies innovate and the role of the ecosystem in the innovation process to understand the innovation framework. Chinese internet companies have become the world’s competitive industry due to their innovation-based competitiveness. However, it is noticed that the growth and development of internet companies is unbalanced around the world. Hence, it is worth examining the innovation in Chinese internet companies to capture the true picture of the industry performance and the role of the ICT ecosystem to understand whether this innovation is enterprise-led or interdependent.

In this investigation, we measure the change in the production function of the industry to estimate the innovation. For this purpose, the technical efficiency of internet companies on a yearly basis using Data Envelopment Analysis (DEA) has been estimated to obtain production function. In the next stage, Meta-frontier analysis (MFA) is applied to compare the production function of the internet industry across the years. Technology Gap Ratio (TGR) is used to analyze the innovation of internet companies using DEA and MFA results. In previous studies, technical efficiency (TE) was utilized to estimate the efficiency of companies where lower TE value is considered an inefficient industry. While this study argued that a decrease in TE value and an increase in TGR value contended that the production functions have moved upward. The upward shift of production functions is due to the innovation of some companies. However, imitation of innovation by other companies defuses the innovation effect and the TE value starts increasing.

## Materials and methods

This study utilized the production function approach to examine innovation in the internet industry. The production function is estimated by measuring the TE of internet companies on a yearly basis. The year-wise efficiency enables us to analyze the evolution and innovation of internet companies. The data envelopment analysis (DEA) has been utilized to estimate the efficiency of internet companies each year. The companies in the same industry are diverse due to different technologies. However, it is assumed that the production function of companies in an industry is similar. Further, Meta-frontier analysis (MFA) has been estimated to compare the production function of the internet industry each year [21]. The meta-frontier analysis (MFA) has been introduced by [22] to envelop traditional production function. Traditionally MFA was applied in the agriculture industry to compare agriculture productivity and efficiency across different groups (countries, regions, etc.) [23]. The scope of MFA application has surged recently into many areas including dairy farming, civil engineering, energy, banking and pharmaceutical sectors [24–27]. Furthermore, recent trends in the meta-frontier applications have enclosed the astonishing results in the ICT industry as reported by [28,29].

### Model estimation

This study utilized DEA based meta-frontier analysis to determine the efficiencies of internet companies. DEA is a useful (deterministic) technique to measure technical efficiency (TE) for a homogeneous group. Efficiency can be depicted as the ability to deliver a specific yield with a given input level by downplaying the wastage level. Effectiveness estimation is significant in decreasing asset to squander and performance enhanced [30]. Very few studies have been found in this regard to assess the efficiency of internet companies through DEA. The use of DEA to measure the efficiency of internet companies was introduced by Baura [31]. Later, Serrano Cinca [18] has applied the DEA method to estimate the efficiency of dot com companies. The approach of DEA is to build up an efficient frontier to exhibit the most ideal yield for a certain input level [32,33]. The distance of observations from the efficient frontier explains the efficiency level where observations lie on the frontier are considered fully efficient and vice -versa for observations lie underneath the efficient frontier. [34]. Two DEA technologies have been introduced known as an input-oriented model and output-oriented model. Both the measurement approaches produce the same frontier while the input-oriented model focuses to obtain minimum input level for a given output level and the output model focuses on maximum output level for a given input level. It is argued that companies have heterogenous production functions due to different production technologies, structure and governing mechanisms hence we adopted a variable return to scale technology [21].

**Fig 1:**
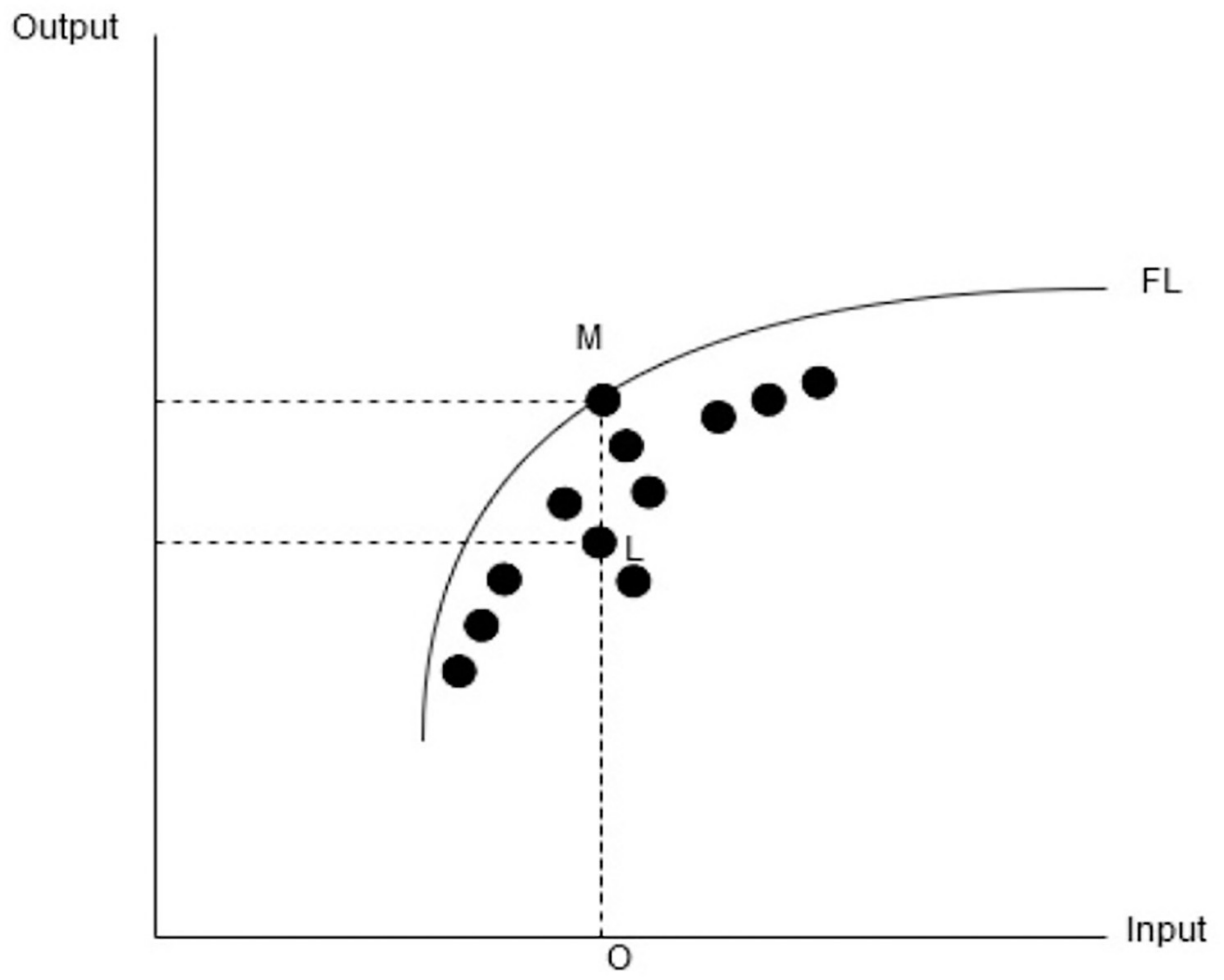
Frontier production function (DEA).

For instance, a homogeneous group of DMU’s as denoted by black dots in the above Fig 1 using input and output values. Though, in real-world firms have more than one input and output variables hence, input and output variables do not have one to one relation. The frontier line (FL) considered maximum efficiency and the firm located on the frontier line is considered fully efficient. In our example, the firm “M” is located on the frontier and considered as fully efficient. The efficiency of a firm located below the frontier line is calculated by the ratio of a firm to the frontier line with the same input level. Hence, the technical efficiency of firm “L” in our example can be calculated as OL/OM.

The selection of the DEA model is based on the situational factors that affect the production and operational decisions. In this study, we applied an input-oriented model because controlling the inputs are considered an easier task for organizations while outputs are difficult to control due to market forces such as new entrants, intense competition and technology [35].

The mathematical model for this study was adapted from [34] and [36] called the Variable Return to Scale (VRS) input-oriented model. Following equation utilized to estimate technical efficiency.

$$\begin{array}{l} \underset{\lambda_{ji}\Phi_{ji}}{min}\Phi_{ji}\\ s.t.\;Y\lambda_{it}-y_{it}\geq 0,\\ \Phi_{it}x_{it}-X\lambda_{it}\geq 0,\\ j^{\prime }\lambda_{it}=1\;\;\text{and}\\ \lambda_{it}\geq 0 \end{array}$$ (1)

Where ***Φ***_***ji***_ represents the scaler inverse of efficiency, the input vector (N × 1) and output vector (M× 1) for ***i***th internet firm in the ***t***th time period is represented by ***x***_***it***_ and ***y***_***it***_ respectively. ***λ***_***it***_ (LT × 1) is the decision variable represents the vector of weights calculated from data. Y (M× LT) and X (N× LT) are the matrices of all input and output quantities for all L firms in all T periods.

The input-orientated technical efficiency (TE) measured with respect to the group frontier for each year is estimated using Eq (1). The meta-frontier (TE*) efficiency using all the data in the sample has been estimated using the same Eq (1).

[36] stated that the group frontier envelops in meta-frontier function, hence we can measure the distance of group frontier with meta-frontier. Therefore, the technology gap ratio can be defined as

$$TGR_{it}=\frac{TE_{it}^{*}\left(x,y\right)}{TE_{it}\left(x,y\right)}$$ (2)

The meta-frontier technical efficiency (TE*) is the product of technical efficiency (TE) and technology gap ratio (TGR). Henceforth Eq (2) can be decomposed as mentioned in Eq (3) based on [36]. This shows that meta-frontier efficiency (TE*) can be easily estimated using Eq (3)

$$TE_{it}^{*}=TE_{it}\times TGR_{it}$$ (3)

**Fig 2:**
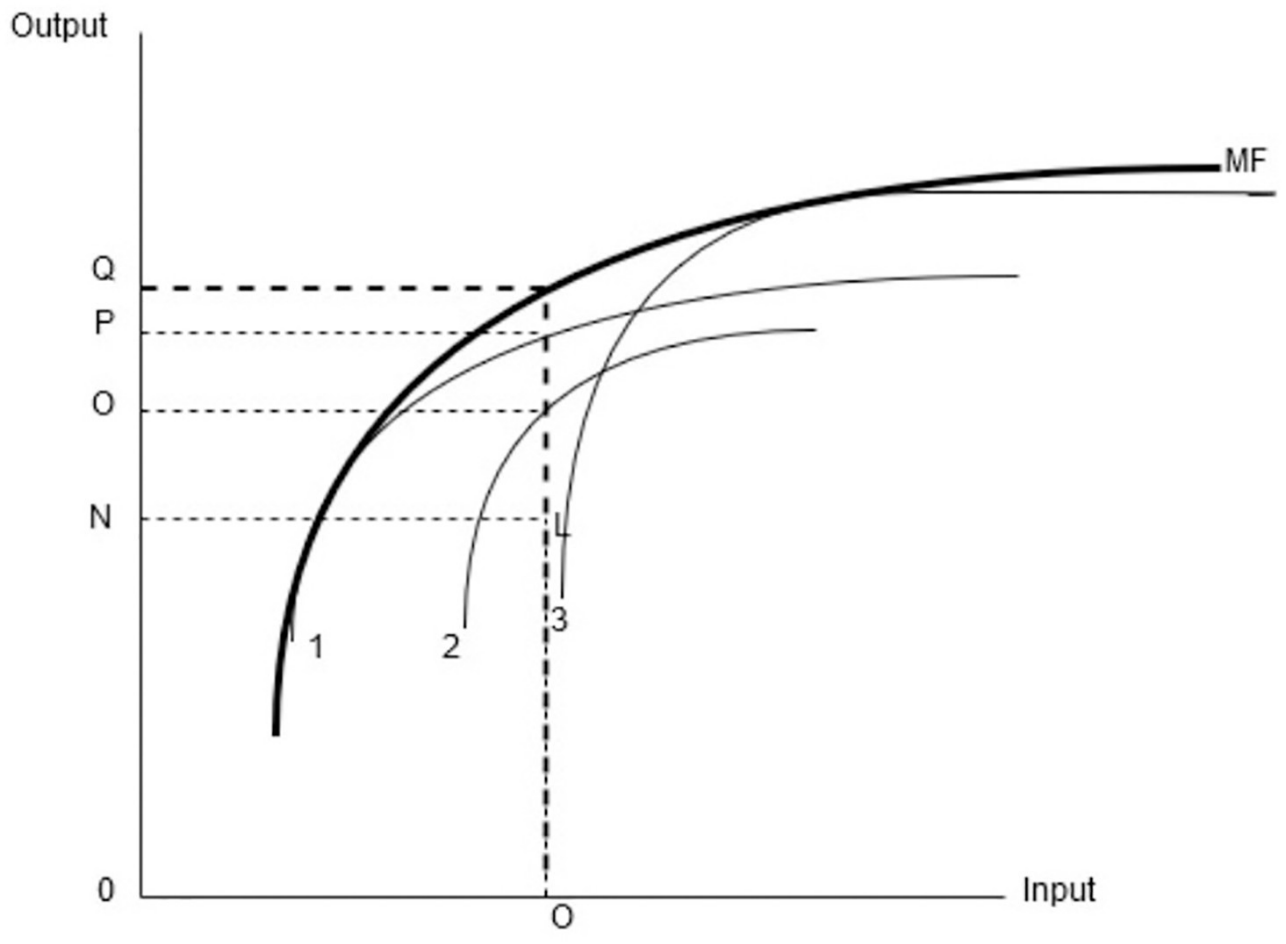
Meta frontier production function (adopted from Battese and Rao, 2002).

In Fig 2 the meta frontier production function is expressed by the MF line which represents the maximum efficiency and group frontiers are denoted as 1, 2 and 3. The meta efficiency (TE*) for firm “L” is 0N/0O. Similarly, the TGR value is the ratio of distance among group frontier and meta frontier (0N/0Q). The DMU’s would be more efficient in group efficiency in contrast with Meta frontier efficiency due to the reason that Meta frontier envelops all the group frontiers [36]. The meta-frontier efficiency for all periods can be estimated by applying a similar DEA model to the whole data sources.

**Fig 3:**
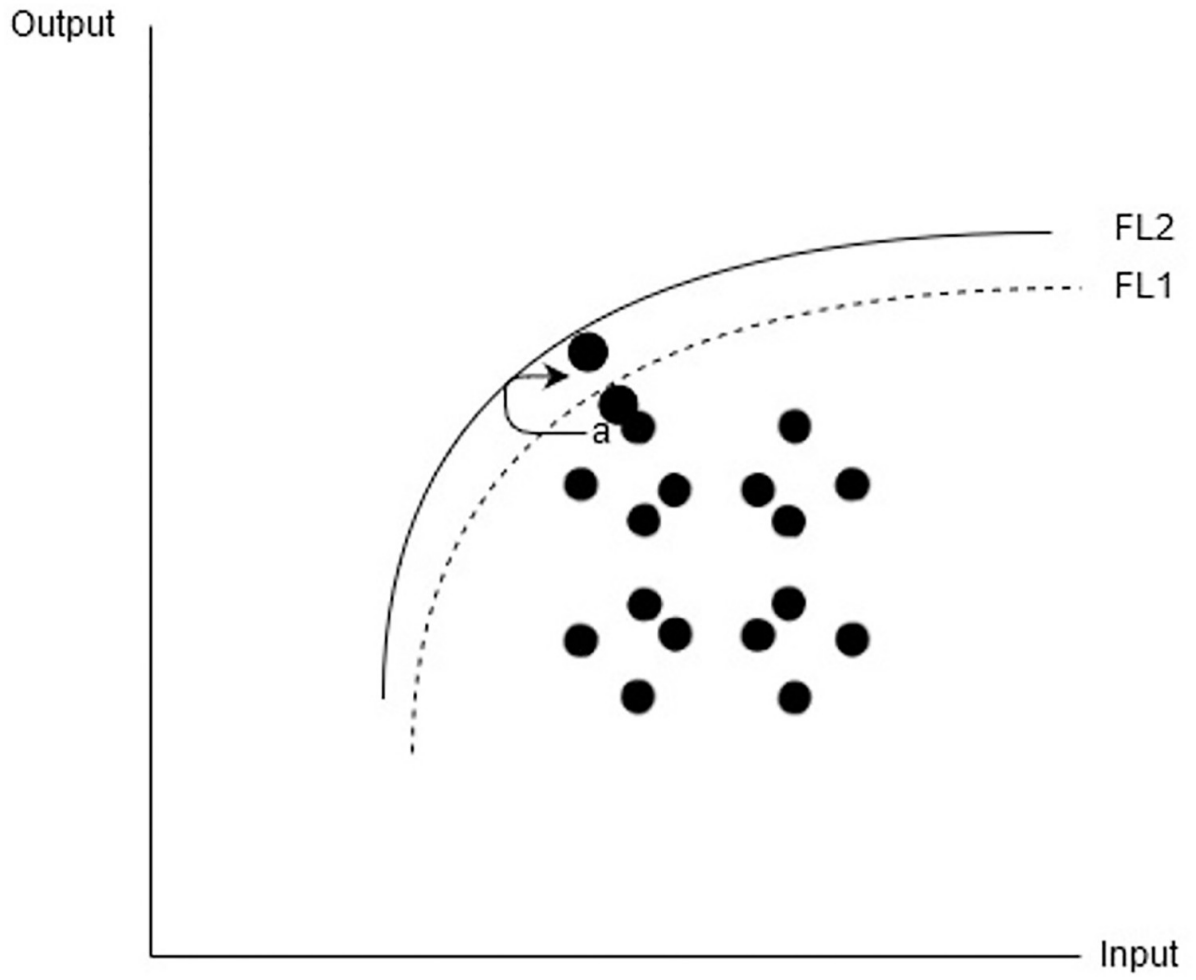
Innovation in industry.

The efficiency measured by the DEA model is the relative efficiency and estimated by calculating the distance from the production frontier. For instance, a company (a) in Fig 3 innovates, the output level of the company significantly increased hence the production frontier shifted upward from FL1 to FL2. Hence, the efficiency of the remaining companies will decrease due to an increase in the distance from the production frontier. As a result, the overall efficiency of the industry will decrease. This shift in the production frontier and the change in the average efficiency of industry or group can be estimated by the technology gap ratio (TGR). The upward shift of the production frontier will decrease the distance between group frontier and meta-frontier hence technology gap ration will increase.

**Fig 4:**
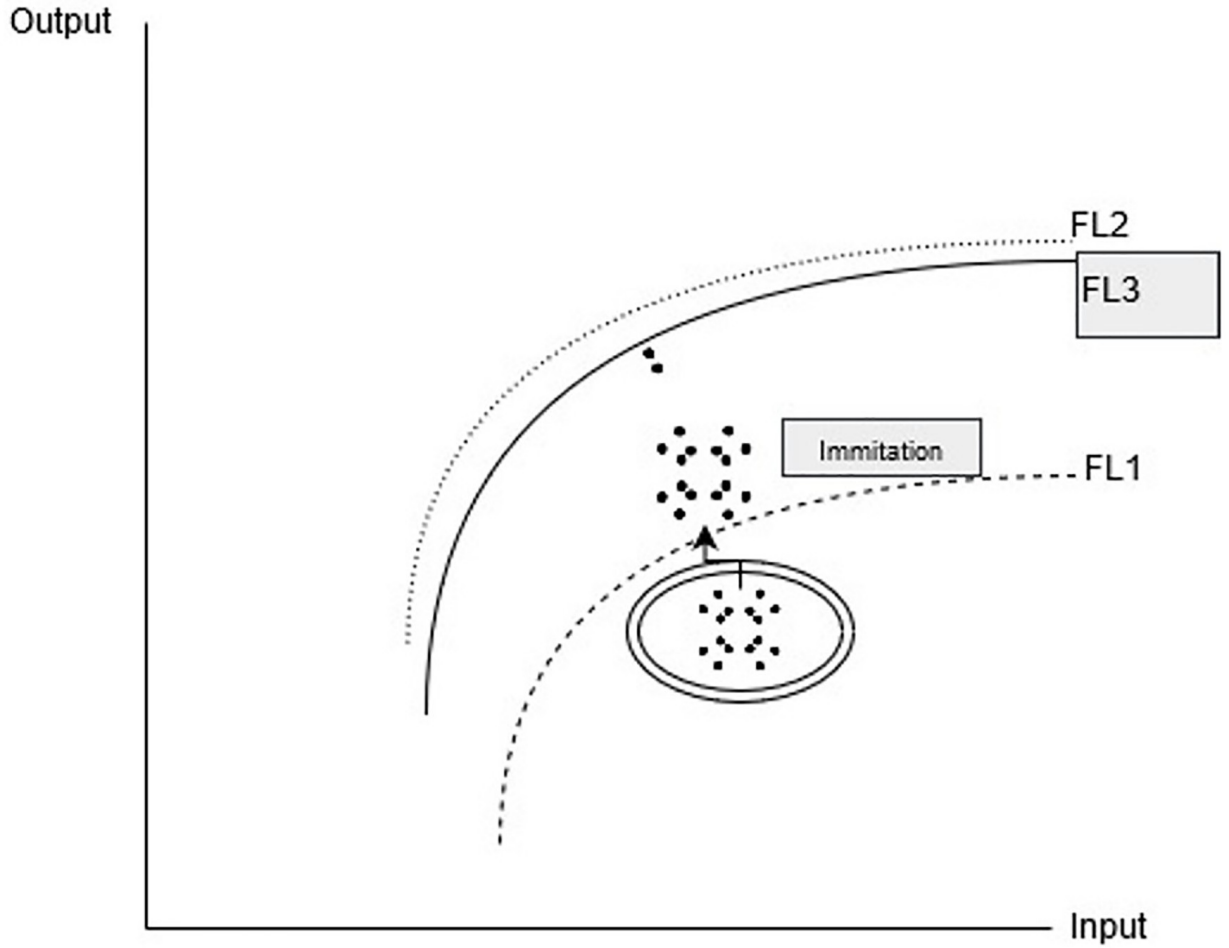
Imitation in industry.

The innovation of a company in an industry will instigate remaining companies to innovate or imitate to remain competitive in the industry. For instance, the imitation of other companies (Fig 4) will raise their efficiency values consequently increase the overall efficiency of the industry. However, it is important to mention that the increase in efficiency values of imitating companies are still below the production frontier. This may decrease the production frontier and the TGR may fall because of the increase in group frontier and meta-frontier. The innovative company will lose competitive advantage and its efficiency value will decline as the efficiency measure is relative in nature. This process of innovation and imitation will continue and the industry will continue to grow. Keeping in view the competitive environment of internet companies, sudden and sharp changes in efficiency values and production function is expected.

## Results and discussion

### Sample and variables

The unbalanced pool of data for the selected variables of Chinese internet companies has been collected from 2000 to 2017 as this era is dominated by the intensive growth of Chinese internet companies. The data set was obtained from the Wind database and DataStream database which are authentic sources of financial data. Additionally, only internet companies indexed in the stock market and over the counter market were selected in the sample. For this study, 325 Chinese internet companies were selected to collect relevant data to measure production function. For inclusion criteria, Internet companies fall in the category of platform, content and application providers [8,9] such as portals & search, content & communities and e-tailers [18] has been selected for this study. For exclusion criteria, the firms were dropped from the sample if any of the input or output variables have zero or negative value.

The selection of input and output variables has been a point of discussion in the previous literature [37,38]. In the DEA model, various factors mix can deliver diverse productivity scores. Therefore it is imperative to choose appropriate input and output variables for effectiveness estimation. Based on [18] contention this study utilized the conception that a firm utilized its staff and assets and make an expenditure to achieve its objective which is revenue. Therefore, three input variables no. of employees (I), capital expenditure (M), and total asset (K) have been selected. The no. of employees was chosen because it is an essential element of production. Capital expenditure represents the cost of the revenue and consistent with prior studies as capital investments provide innovation potential [39]. Total assets represent the resources of the business and include all assets current, fixed and intangible assets. The selected output is revenues measured through total sales (Y). Total revenue is a typical measure in DEA literature [40]. The selected companies in this study are the companies that publish their financial data. Although, companies that do not publish their financial information to the public are excluded from the data set. It is noted that the industry is dominated by big giant companies. Hence, the exclusion of companies that do not publish financial information is considered as small companies and may not affect the significance of the study.

### Data analysis

The input and output variables are summarized in Table 1. Mean and standard deviation values of input and output variables are presented against each year. Where standard deviation is reported in parentheses. Mean total sales is ¥ 518,054,105.6 in 2000 which increased to ¥ 6,113,788,744.7 in 2017. Similarly, the numbers of employees have increased from 2,771 in 2000 to 3,483 in 2017 with a standard deviation of (4337.05) and (11,061.5) respectively. The total assets were 1,717,150,289.7 in 2000 increased to 10,155,260,625.1 in 2017 shows a huge increase in the input and output quantities of internet companies.

**Table 1:** Descriptive statistics of data (¥).

Year	M	Y	K	I
**2000**	86,822,978.05	518,054,105.6	1,717,150,289.7	2,771.07
	(34490079.9)1	(2090985129)	(2455994154)	(4337.05)
**2001**	71,803,681.4	543,328,883.4	1,085,153,566.3	1,342.02
	46,222,683.6	(655,146,417.9)	(329,548,759.7)	(1,111.4)
**2002**	59,867,249.2	662,737,677.04	1,188,140,900.4	1,612.7
	(76304932.12)	(799456943.2)	(1356527964)	(1403.6)
**2003**	63,083,515.2	695,194,293.4	1,221,915,968.5	2,064.5
	(79091694.22)	(848190701.4)	(1369392083)	(2801.7)
**2004**	49,808,550.5	652,094,958.2	1,149,624,806.5	1,857.6
	(71971207.91)	(814695346.4)	(1335479246)	(2512.8)
**2005**	49,123,417.2	662,707,114.06	1,025,342,014.5	1,627.7
	(65371160.71)	(728270135.1)	(1052654752)	(1987.3)
**2006**	45,931,396.9	841,901,772.8	1,871,596,499.1	1,670.5
	(97801777.19)	(2240835113)	(9379931091)	(2511.0)
**2007**	68,898,027.2	858,074,685.1	2,355,403,239.7	1,575.7
	(166,152,440.4)	(2,784,379,261)	(11,003,384,121.5)	(2,419.5)
**2008**	64,920,757.1445	826,508,023.1820	1,220,351,310.1090	1,537.5
	1(79,514,645.5)	(2,326,875,341.3)	(2,536,658,042.9)	(2,508.1)
**2009**	53,308,262.1286	807,906,753.4293	1,070,497,007.0125	1,701.2
	(118,480,052.9)	(1,674,045,038.1)	(1,966,241,214.1)	(2,844.3)
**2010**	110,396,711.8759	986,330,708.4601	1,472,052,685.1927	1,844.3
	(242,253,742.7)	(2,360,352,681.6)	(3,337,270,555.6)	(2,892.2)
**2011**	138,310,633.0590	1,369,431,448.5486	2,303,843,018.8258	2,109.2
	(440,393,384.2)	(3,734,587,544.3)	(6,257,100,509.8)	(3,516.7)
**2012**	135,205,087.7	1,702,329,906.4	2,452,080,722.8	2,469.9
	(439,044,769.2)	(5,425,749,451.8)	(7,728,343,419.5)	(4,381.6)
**2013**	133,880,507.4	1,793,092,516.4	2,675,572,978.4	2,310
	(550,020,490.6)	(7,035,432,378.6)	(10,651,933,936.7)	(4,896.9)
**2014**	176,922,202.0	2,242,037,586.8	3,805,519,483.4	2,587.1
	(798,375,895)	(9,896,042,787.4)	(19,342,765,404)	(6,611.9)
**2015**	250,348,125.1	3,174,859,712.4	5,810,294,194.7	2,997.7
	(1,180,415,011.3)	(14,185,966,327.7)	(29,684,044,252)	(8,515.9)
**2016**	318,373,865.6	4,331,778,990.7	7,623,462,932.8	3,221.2
	(1,651,225,687.4)	(20,071,819,357.2)	(39,074,271,382.1)	(9,089.3)
**2017**	443,120,694.8	6,113,788,744.7	10,155,260,625.1	3,483.1
	(2,719,090,428.5)	(29,524,847,199.8)	(54,394,485,905)	(11,061.5)

Table 2 below presents the average TE values obtained from data envelopment analysis in each year. Further, the technology gap ration (TGR) is calculated as the ratio of Meta frontier efficiency and technical efficiency and presented in Table 3. Meta frontier efficiency is presented in Table 4. In the final section, we analyze the moment and changes in three indices to capture the innovation mechanism in internet companies.

**Table 2:** TE values obtained from DEA.

Year	Mean	S.D	Maximum	Minimum
**2000**	0.7027	0.08913	1	0.08161
**2001**	0.7186	0.08128	1	0.10375
**2002**	0.7225	0.0634	1	0.09712
**2003**	0.7161	0.09675	1	0.12729
**2004**	0.7106	0.08343	1	0.10120
**2005**	0.7081	0.08122	1	0.09682
**2006**	0.7217	0.07847	1	0.05682
**2007**	0.7241	0.09167	1	0.04041
**2008**	0.6923	0.10591	1	0.01243
**2009**	0.6901	0.10581	1	0.01769
**2010**	0.7102	0.10469	1	0.06079
**2011**	0.713	0.15010	1	0.01119
**2012**	0.7177	0.14545	1	0.02512
**2013**	0.7136	0.14541	1	0.01879
**2014**	0.7112	0.14815	1	0.09482
**2015**	0.7085	0.14471	1	0.09588
**2016**	0.7058	0.11870	1	0.07519
**2017**	0.7091	0.16651	1	0.06964

**Table 3:** TGR values of Chinese internet companies.

Year	Mean	S.D	Maximum	Minimum
**2000**	0.8957	0.1832	1	0.1362
**2001**	0.8770	0.2376	0.9726	0.0824
**2002**	0.8839	0.1938	0.9821	0.0625
**2003**	0.8893	0.1725	0.9881	0.1104
**2004**	0.9050	0.1981	0.9982	0.0930
**2005**	0.9139	0.1612	1	0.0977
**2006**	0.8904	0.2963	1	0.1061
**2007**	0.8950	0.1383	0.8922	0.0421
**2008**	0.9383	0.2579	1	0.0317
**2009**	0.9415	0.2565	1	0.0885
**2010**	0.9164	0.1823	0.8732	0.0946
**2011**	0.9316	0.2046	0.9207	0.0213
**2012**	0.9289	0.1984	1	0.0491
**2013**	0.9351	0.2110	1	0.0730
**2014**	0.9391	0.2212	0.9810	0.0263
**2015**	0.9431	0.1821	1	0.0414
**2016**	0.9449	0.2263	1	0.0493
**2017**	0.9466	0.2438	1	0.0441

**Table 4:** TE* values obtained from MFA.

Year	Mean	S.D	Maximum	Minimum
**2000**	0.6294	0.1332	1	0.1362
**2001**	0.6302	0.1474	0.9726	0.0824
**2002**	0.6386	0.1357	0.9821	0.0625
**2003**	0.6368	0.1425	0.9881	0.1104
**2004**	0.6431	0.1467	0.9982	0.0930
**2005**	0.6471	0.1599	1	0.0977
**2006**	0.6426	0.1579	1	0.1061
**2007**	0.6481	0.1659	0.8922	0.0421
**2008**	0.6496	0.1715	1	0.0317
**2009**	0.6497	0.1787	1	0.0885
**2010**	0.6508	0.1826	0.8732	0.0946
**2011**	0.6642	0.1890	0.9207	0.0213
**2012**	0.6667	0.1924	1	0.0491
**2013**	0.6673	0.2302	1	0.0730
**2014**	0.6679	0.2391	0.9810	0.0263
**2015**	0.6682	0.2481	1	0.0414
**2016**	0.6669	0.2408	1	0.0493
**2017**	0.6712	0.2675	1	0.0441

The change in average TE and TGR values over the period of study helps to analyze the innovation in the industry and change in the production function. Table 2 shows the change in TE value over the period of study. It is noted that TE values decrease in 2003–05, 2008–09 and trend continue from 2013 till 2017. The overall trend in TE values shows ups and down which contented the inconsistent performance of the internet industry. However, if we combine the change in TE values with the change in TGR values (Table 3), the results revealed interesting facets.

In contrast with TE values, TGR values (Table 3) are increasing in 2003–05, 2008, 2009 and its increasing trend continues from 2013 to 2017. This contented that the decrease in TE value (Table 2) and an increase in the TGR value of internet companies at the same time is not due to the inefficiency of some internet companies. Rather, the innovation by some internet companies has moved the production function upward. This shift in production function decreases the distance of group frontier with meta frontier hence, TE decreased.

Similarly, the trend in TE* in Table 4 shows that there is a steady increase in meta-frontier efficiency. This increase in meta-frontier efficiency shows that the industry is growing over the period of study.

**Fig 5:**
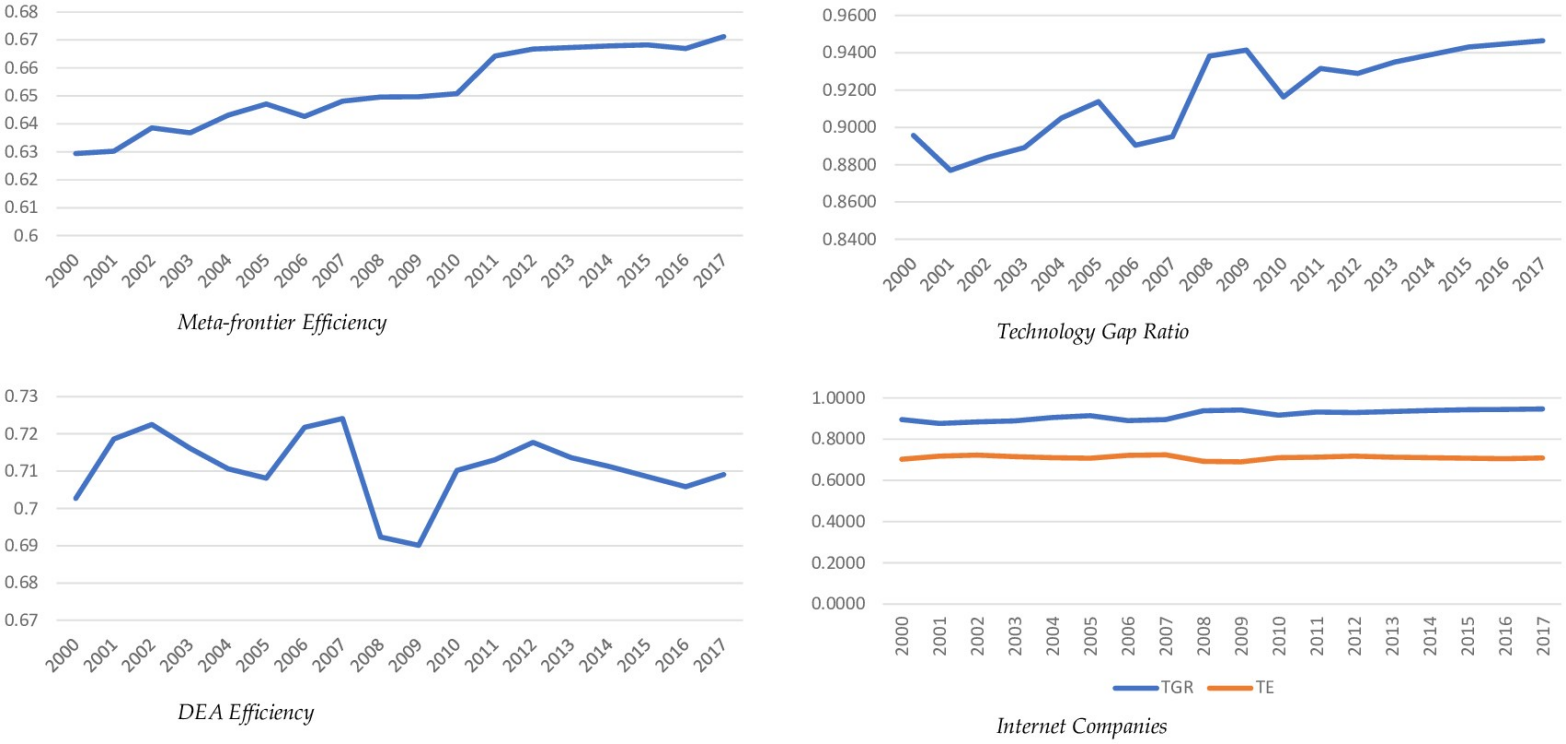
Change in TE, TGR, and TE* in internet companies by year.

Fig 5 explains and concludes the overall pattern of change in technical efficiency, meta frontier efficiency, and technology gap ratio. As we can see the increase in technical efficiency brings a similar decrease in technology gap ratio and vice versa. While meta-frontier efficiency is increasing steadily.

When we combine the TE, TE* and TGR trajectory with the technological changes in the ICT ecosystem, we found the factors contribute and support the innovation in internet companies. As the TE value decrease from 2003 to 2005 and the TGR value increases, the distance between meta-frontier production function and group frontier production function increased. This increased distance is due to the innovation of firms that grab the opportunity of the emergence of broadband internet. The emergence of the personal computer market with broadband internet in China makes a new shift in the industry with dot com boom. Similarly, the change in TE and TGR values in 2008 and 2009 is again due to the first-mover advantage of some companies that emerged with the launch of smartphones by Apple in 2007. In addition to this, a surged has started in online gaming which makes China the world’s biggest market in online gaming users in 2009 (China Internet Network Information Center, January 2013). Hence, internet companies adopted an innovation-led business model by adopting emerging technologies and compliance with the new ICT ecosystem [8] and moved the overall production function upward. The imitation of innovation decreases the TGR value and average TE increased as the first-mover advantage diffused.

Since 2014 the TGR value is steadily increasing while TE values are continuously decreasing which shows that the production function is moving upward continuously. The adoption of artificial intelligence and mobile payment system provide innovation opportunities for internet companies particularly e-commerce industry. The continuous increase in TGR value shows that the innovation-led growth of some companies adopted sustainable innovation-based business models. Furthermore, strategic business alliances of big internet companies have created a huge difference in meta-frontier production function and group production function. This makes it difficult for other companies to imitate the business model hence the overall TE of the internet industry is decreasing while TGR is increasing at the same time.

Our study provides useful insights into the innovation of internet companies and the role of external factors that contribute to the innovation of the internet industry in China. Our findings observe the behavior of innovation in internet companies and linked external events to explain the innovation paradox of Chinese internet companies. The result of our study shows that there is a sharp decrease and large variances in mean TE values on which one may assume that all the firms are inefficient and industry productivity has decreased. However, we argued in contradiction with previous studies [16–18,41] by introducing the different methodological approach. It is noted that the decrease in TE value is not due to the inefficiency of internet companies while the production function is lifted upward due to the innovation of some companies as explained by an increase in TGR value at the same time. Further, a steady increase in meta efficiency (TE*) of internet companies contented that the industry is growing over the period of study. Moreover, the decrease in TE* value when TGR is increasing revealed that the industry has higher growth potential in that particular year compared to other years.

## Conclusions

Innovation is not only significant to achieve organizational success but also significant for the development of the industry. The overwhelming growth of Chinese internet companies stimulates the authors to analyze the evolution and innovation process. As a part of the ICT ecosystem, internet companies have not developed on their own. Therefore, it is worth investigating whether the production function of the internet industry is enterprise-led innovation, or affected by external factors. However, sustainable innovation-based business model deemed as a key driver for business development in the ever-changing ICT landscape [4].

This investigation used a DEA based meta-frontier method to measure the innovation in Chinese internet companies. Whereas, in previous researches, DEA is used to measure the production function by estimating TE (technical efficiency) and the decreasing value of TE was considered an inefficient industry. However, in this investigation, we calculate TGR using meta-frontier analysis to measure the technology gap. It is noted that as the average TE value decreased in a particular year, the TGR value increased in response. This contends that the decrease in average TE and increase in TGR is due to the innovation of some companies which move the production frontier upward. As the innovation is imitated by the other internet companies the first-mover advantage neutralized and average TE starts increasing while TGR starts decreasing. In addition to this, the pattern of innovation over the period of the study revealed that innovation in internet companies is not solely business-led but development in the ICT ecosystem landscape provides opportunities for the internet companies to innovate. Further, the continuous increase in TGR since 2013 revealed that some internet companies have adopted a sustainable innovation-led business model which is moving the production function upward. The distance between group frontier (year) and meta-frontier are increasing which indicates that firms are moving group production function through innovation. Therefore, a steady increase in TE* understood as the industry has growth potential. This investigation provides significant results to understand and support innovation in the Chinese internet industry. It is recommended that policymakers should understand the ICT ecosystem and develop better strategies to support the ICT ecosystem as a whole. As the layers of the ICT ecosystem have a symbiotic relationship therefore, innovation in one layer will affect the other layers. Hence, a collaboration among different layers of the ICT ecosystem will create more opportunities for innovation which will make the ICT industry more competitive. This study concludes that a well establish and collaborative ICT environment is necessary to promote innovation where the co-evolutionary mechanism in the ICT industry can be promoted with national support. Countries that are lacking behind in the internet industry need to realize the characteristics of their ICT ecosystem while making policies to facilitate and strengthen the ICT ecosystem.

Our study is limited to internet companies in China which is only one layer of the ICT ecosystem. Whereas, the layers of the ICT ecosystem have a co-evolutionary mechanism, therefore, it is important to investigate the innovation in other layers to capture the collective effect. Furthermore, regulations and socio-economic conditions are crucial while analyzing the innovation of companies hence, this framework needs further verifications in different social and work settings.

## Supporting information

S1 Dataset (XLSX)
